# *ANGPTL4* variants and their haplotypes are associated with serum lipid levels, the risk of coronary artery disease and ischemic stroke and atorvastatin cholesterol-lowering responses

**DOI:** 10.1186/s12986-018-0308-5

**Published:** 2018-10-05

**Authors:** Qian Yang, Rui-Xing Yin, Xiao-Li Cao, Feng Huang, Yi-Jiang Zhou, Wu-Xian Chen

**Affiliations:** 1 Department of Cardiology, Institute of Cardiovascular Diseases, the First Affiliated Hospital, Guangxi Medical University, Nanning, 530021 Guangxi People’s Republic of China; 2 Department of Neurology, the First Affiliated Hospital, Guangxi Medical University, Nanning, 530021 Guangxi People’s Republic of China

**Keywords:** Angiopoietin-like protein 4 gene, Single nucleotide polymorphism, Coronary artery disease, Ischemic stroke, Lipids, Atorvastatin

## Abstract

**Background:**

This study aimed to assess the association between the angiopoietin-like protein 4 gene (*ANGPTL4*) single nucleotide polymorphisms (SNPs) and serum lipid levels, the risk of coronary artery disease (CAD) and ischemic stroke (IS), and response to atorvastatin therapy in a Southern Chinese Han population.

**Methods:**

Genotypes of the *ANGPTL4* rs4076317, rs7255436, rs1044250 and rs2967605 SNPs in 1,654 unrelated subjects (CAD, 568; IS, 537; and controls, 549) were determined by the Snapshot technology. Another group of 724 hyperlipidemic patients was selected and treated with atorvastatin calcium tablet 20 mg/day for 8 weeks.

**Results:**

The rs2967605 CT/TT genotypes were associated with a decreased risk of CAD (adjusted OR = 0.68, 95% CI = 0.47-0.99, *P* = 0.043 for CT/TT *vs*. CC) and IS (adjusted OR = 0.55, 95% CI = 0.38-0.80, *P* = 0.020 for CT/TT *vs*. CC). There was no significant association between the four SNPs and angiographic severity of CAD. The subjects with the rs4076317 CG/CC genotypes in controls had higher total cholesterol (TC) and low-density lipoprotein cholesterol (LDL-C) levels than the subjects with the GG genotype (*P* < 0.001; a *P* < 0.0018 was regarded statistically significant by the Bonferroni correction). The subjects with rs4076317CG/GG genotypes had lower TC and LDL-C levels than the subjects with CC genotype after atorvastatin treatment (*P* < 0.001).

**Conclusions:**

The observed associations suggest that the *ANGPTL4* variants have a potential role on serum lipid levels and atherosclerosis-related diseases in the Chinese Han population, especially the *ANGPTL4* rs4076317 and rs2967605 SNPs.

## Background

Atherosclerosis development is closely associated with lipid disorders [1–5]. The retention of serum lipoproteins in the artery wall is a key initiator of atherosclerosis [6]. The accumulation of oxidized lipoproteins in the artery wall sets off a cascade of proinflammatory events leading to the recruitment of macrophages, lipids uptake into these cells, and the initiation of the chronic inflammatory cascade that characterizes atherosclerosis [6]. Thus, dyslipidemia plays critical roles in the initiation and progression of the atherosclerotic lesion. In recent genome-wide association studies (GWASes), multiple lipid-related loci have been identified, which is important to unravel novel pathways and their relations to atherosclerosis-related diseases, so that provided more effective means of diagnosis, treatment, and prevention for atherosclerosis [7, 8].

Angiopoietin-like protein 4 gene (*ANGPTL4*) is one of the novel genes associated with serum lipid levels in the Caucasian population [7, 8]. The human *ANGPTL4* is located on chromosome 19p13, which contains seven protein-coding exons and two non-coding exons, and encodes a 406-amino-acid glycoprotein with a molecular mass of 50 kDa [9, 10]. *ANGPTL4* also known as hepatic fibrinogen angiogenic related protein, fasting induced adipose factor, and peroxisome proliferators-activated receptor (PPAR) angiogenic related protein, and these names collectively give insight into the expression and function of the protein [11, 12]. Previous functional studies revealed that *ANGPTL4* regulated plasma triglyceride (TG) levels by inhibiting lipoprotein lipase (LPL) [13–16]. LPL is responsible for catalyzing the hydrolysis of TG in chylomicrons and very low-density lipoproteins (VLDLs) and enhancing the high-density lipoprotein cholesterol (HDL-C) levels, and regulating the supply of fatty acids to various tissues for either storage or oxidation [17, 18]. *ANGPTL4*-deficient mice decreased serum TG levels by increasing LPL activity. In contrast, the *ANGPTL4* transgenic mice elevated serum TG and reduced LPL activity [14, 15, 19–21].

Some studies have also showed that *ANGPTL4* loss-of-function mutations are associated with substantially lower TG levels, and a lower risk of coronary artery disease (CAD) and type 2 diabetes (T2D) [22–25]. Furthermore, *ANGPTL4* has been considered to be a promising drug target for therapeutic intervention against hyperlipidemia and atherosclerosis-related diseases. However, there is no universal agreement on the association between the *ANGPTL4* variants and serum lipid traits, and the risk of CAD and ischemic stroke (IS) in different populations. Therefore, the purpose of the present study was to assess the association between 4 *ANGPTL4* SNPs (rs4076317, rs7255436, rs1044250 and rs2967605) and serum lipid levels, the risk of CAD and IS, and the lipid-lowering efficacy of atorvastatin in a Southern Chinese Han population.

## Methods

### Study samples

The present study enrolled 1,105 unrelated patients with CAD (*n* = 568) and IS (*n* = 537) from hospitalized patients in our First Affiliated Hospital. The diagnosis of CAD was based on typical clinical symptoms and electrocardiographic changes, as well as increases in the serum markers including creatinine kinase-MB and troponin T. Coronary angiography was performed in all CAD patients. Only significant coronary stenosis with lumen narrowing (≥ 50%) in at least either one of the three main coronary arteries or their major branches (branch diameter ≥ 2 mm) was selected. Additionally, the angiographic severity of disease was defined as single or multi-vessel disease based on the number of involved artery (luminal narrowing ≥ 50%) in the three major coronary arteries [26, 27]. All of the IS patients received a strict neurological examination and brain magnetic resonance imaging (MRI). The classification of IS was made according to the Trial of Org 10172 in Acute Stroke Treatment (TOAST) criteria [28]. The selected IS patients included individuals who were eligible for one of the two subtypes of TOAST criteria: Large-artery atherosclerosis and small-vessel occlusion. Subjects with a history of hematologic, neoplastic, renal, liver, thyroid, autoimmune diseases and type I diabetes mellitus were excluded. The CAD patients who had a past history of IS, or the IS cases who had a past history of CAD were excluded from the study. There were 56 patients not included in this study because of the co-existence of both CAD and IS.

In addition, the present study also enrolled 549 control subjects matched by age, gender, and ethnic group (Han Chinese) from Physical Examination Center of our First Affiliated Hospital during the same period. The controls were free of IS and CAD by history taking, clinical, biochemical, and image examinations such as 64-slice computed tomographic coronary angiography. The study design was approved by the Ethics Committee of the First Affiliated Hospital, Guangxi Medical University (No: Lunshen-2011-KY-Guoji-001; Mar. 7, 2011). Informed consent was obtained from all participants.

### Atorvastatin treatment group

Another group of 724 hyperlipidemic patients (controls, 253; CAD, 248 and IS, 223) was also enrolled and treated with atorvastatin calcium tablet (Lipitor, Pfizer Wuxi Pharmaceutical Co., Ltd.) 20 mg per day for 8 weeks after the genotype identification. The vast majority of them were selected from the above study samples, and a few subjects were new diagnostic cases. There were 420 men (58%) and 304 women (42%). The ages ranged from 34 to 76 years, with an average age of 60.18 ± 12.35 years. The individuals who had taken lipid-lowering drugs such as statins or fibrates in two weeks were not included in this group. The study protocol was also approved by the Ethics Committee of our First Affiliated Hospital. All patients signed an informed consent form. The individuals with total cholesterol (TC) > 5.17 mmol/L, and/or TG > 1.70 mmol/L were defined as hyperlipidemic [29–32]. Clinical biochemistry analyses including serum lipid levels were performed before and after 8 weeks of atorvastatin treatment.

### Biochemical measurements

Venous blood sample was obtained from all subjects after at least 12 hours of fasting. The levels of serum TC, TG, HDL-C, and low-density lipoprotein cholesterol (LDL-C) in samples were determined by enzymatic methods with commercially available kits. Serum apolipoprotein (Apo) A1 and ApoB levels were detected by the immunoturbidimetric immunoassay. The normal values of serum TC, TG, HDL-C, LDL-C, ApoA1, ApoB levels and the ApoA1/ApoB ratio in our Clinical Science Experiment Center were 3.10–5.17, 0.56–1.70, 1.16–1.42, 2.70–3.10 mmol/L, 1.20–1.60, 0.80–1.05 g/L and 1.00–2.50, respectively [29–32]. Hypertension was diagnosed according to the criteria of the JNC 7 hypertension guidelines [33]. T2D was diagnosed according to the American Diabetes Association (ADA) criteria for DM in 2012 [34]. Normal weight, overweight and obesity were defined as a body mass index (BMI) < 24, 24–28, and > 28 kg/m^2^; respectively [35].

### SNP selection and genotyping

The rs4076317, rs7255436, rs1044250 and rs2967605 SNPs were selected on the basis of the following assumptions: (1) Selected SNPs were established by Haploview (Broad Institute of MIT and Harvard, USA, version 4.2); (2) Information of the SNPs was obtained from NCBI dbSNP Build 132 (http://www.Ncbi.nlm.nih.gov/SNP/); (3) SNPs were restricted to minor allele frequency (MAF) > 1%. (4) SNPs might be associated with serum lipid levels and the risk of CAD and IS in recent studies [7, 8, 10, 36–38].

Genomic deoxyribonucleic acid (DNA) was extracted from leucocytes of venous blood using the phenol-chloroform method. Genotyping of the four SNPs was performed by the Snapshot technology platform in the Center for Human Genetics Research, Shanghai Genesky Bio-Tech Co. Ltd.

### Statistical analyses

The statistical software package SPSS 21.0 (SPSS Inc., Chicago, Illinois) was used for the statistical analyses. Quantitative variables were expressed as mean ± standard deviation (serum TG levels were presented as medians and interquartile ranges because of non-normal distribution). Qualitative variables were expressed as percentages. Allele frequency was determined via direct counting, and the standard goodness-of-fit test was used to test the Hardy-Weinberg equilibrium (HWE). A chi-square analysis was used to evaluate the difference in genotype distribution and sex ratio between the groups. The general characteristics between patients and controls were tested by the Student’s unpaired *t*-test. The association of genotypes and serum lipid parameters such as TC, HDL-C, LDL-C, ApoA1, ApoB levels and the ApoA1/ApoB ratio was tested by analysis of covariance (ANCOVA; genotypes and TG using Kruskal-Wallis test). Bonferroni correction was employed for variants associated with serum lipid parameters, and a *P* < 0.0018 (0.05/4×7) was considered statistical significant. Unconditional logistic regression was used to assess the correlation between genotypes and the risk of CAD and IS after age, gender, BMI, smoking, alcohol consumption, T2D, hypertension and hyperlipidemia were adjusted. The correlation risk was estimated by odds ratio (OR) and 95% confidence interval (95%CI). The pattern of pair-wise linkage disequilibrium (LD) between the selected SNPs was measured by *r*^2^, and haplotype analyses were performed using the SHEsis software [39]. A two-tailed *P* value less than 0.05 was considered statistically significant for the remaining variables.

## Results

### Characteristics of the study populations

The clinical characteristics of the patients and controls are shown in Table 1. The differences in age, gender, serum LDL-C and ApoB levels, and the percentages of subjects who smoked cigarettes were not significant between controls and patients (*P* > 0.05). As compared with the controls, more CAD/IS patients had T2DM, hypertension and hyperlipidemia; and the CAD/IS patients also had higher BMI, systolic blood pressure, pulse pressure, serum TG levels, the frequency of using lipid-lowering drugs, and lower serum TC, HDL-C, ApoA1 levels, the ApoA1/ApoB ratio and the percentages of subjects who consumed alcohol (*P* < 0.05). There was no difference in diastolic blood pressure levels between controls and IS patients (*P* > 0.05). In comparison with CAD group, the IS patients had lower BMI and higher blood pressure and serum HDL-C levels, and the prevalence of hypertension (*P* < 0.05).

**Table 1:** General characteristics and serum lipid levels between the controls and patients

Characteristic	Control	CAD	IS	*P*_1_	*P*_2_	*P*_3_
Number	549	568	537			
Male/female	384/165	419/149	389/148	0.155	0.364	0.619
Age, years	61.87±11.12	62.23±10.59	62.80±12.41	0.589	0.196	0.408
Body mass index, kg/m^2^	22.28±2.82	23.85±3.37	23.43±3.52	<0.001	<0.001	0.041
Systolic blood pressure, mmHg	130.00±20.60	132.97±23.16	147.58±21.96	0.024	<0.001	<0.001
Diastolic blood pressure, mmHg	82.38±13.01	79.05±14.06	83.71±12.95	<0.001	0.092	<0.001
Pulse pressure, mmHg	49.53±14.92	53.83±17.59	63.83±17.96	<0.001	<0.001	<0.001
Cigarette smoking, n (%)	235 (42.8)	246 (43.3)	224 (41.7)	0.865	0.716	0.592
Alcohol consumption, n (%)	245 (44.6)	132 (23.2)	144 (26.8)	<0.001	<0.001	0.170
Total cholesterol, mmol/L	4.92±1.11	4.51±1.23	4.52±1.14	<0.001	<0.001	0.836
Triglyceride, mmol/L	1.01 (0.64)	1.36 (0.94)	1.35 (0.93)	<0.001	<0.001	0.467
HDL-C, mmol/L	1.90±0.49	1.14±0.34	1.23±0.40	<0.001	<0.001	<0.001
LDL-C, mmol/L	2.74±0.79	2.71±1.01	2.68±0.90	0.549	0.245	0.638
Apolipoprotein (Apo) A1, g/L	1.40±0.24	1.04±0.52	1.02±0.22	<0.001	<0.001	0.608
ApoB, g/L	0.90±0.21	1.11±0.75	0.89±0.25	0.313	0.502	0.302
ApoA1/ApoB	1.63±0.47	1.34±0.46	1.17±0.61	0.009	<0.001	0.117
Type 2 diabetes, n (%)	43 (7.8)	91 (16.0)	81 (15.1)	<0.001	<0.001	0.668
Hypertension, n (%)	157 (28.6)	200 (35.2)	285 (53.1)	0.018	<0.001	<0.001
Hyperlipidemia, n (%)	175 (31.9)	220 (38.7)	238 (44.3)	0.017	<0.001	0.100

*CAD* coronary artery disease, *IS* ischemic stroke, *HDL-C* high-density lipoprotein cholesterol, *LDL-C* low-density lipoprotein cholesterol. The value of triglyceride was presented as median (interquartile range), the difference between CAD/IS patients and controls was determined by the Wilcoxon-Mann-Whitney test. *P*_1_, CAD *vs*. controls; *P*_2_, IS *vs*. controls; *P*_3_, CAD *vs*. IS

### *ANGPTL4* SNPs and the risk of CAD and IS

The genotypic and allelic frequencies of the *ANGPTL4* SNPs are presented in Table 2. The genotype distribution was concordant with the HWE in both cases and controls. Among the four SNPs, only the rs2967605 SNP was shown significant differences in genotype frequencies between the controls and patients (*P <* 0.05). The rs2967605T allele carriers had lower risk of CAD (adjusted OR = 0.68, 95% CI = 0.47-0.99, *P* = 0.043) and IS (adjusted OR = 0.55, 95% CI = 0.38-0.80, *P* = 0.020).

**Table 2:** Effect of the *ANGPTL4* SNPs on the risk of CAD and IS, angiographic severity of CAD

Genotype	Control (% )	CAD (% )	IS (% )	OR (95% CI)_CAD_	*P*_CAD_	OR (95% CI)_IS_	*P*_IS_	OR (95% CI)_AS_	*P*_AS_
rs4076317									
GG	7.3	7.6	7.6	1.00		1.00		1.00	
CG	39.7	39.4	41.0	1.01 (0.61-1.67)	0.971	0.83 (0.50-1.40)	0.493	1.20 (0.58-2.50)	0.621
CC	53.0	53.0	51.4	0.94 (0.56-1.56)	0.797	0.89 (0.53-1.48)	0.656	0.98 (0.47-2.04)	0.947
*P*		0.982	0.867						
*P*_HWE_	0.925	0.882	0.755						
Any C *vs*. GG	92.7	92.4	92.4	0.92 (0.56-1.52)	0.893	0.83 (0.50-1.37)	0.491	1.10 (0.54-2.22)	0.800
Any G *vs*. CC	47.0	47.0	48.6	0.94 (0.72-1.21)	0.611	0.96 (0.74-1.25)	0.778	0.81 (0.56-1.19)	0.289
rs7255436									
AA	1.3	1.2	0.7	1.00		1.00		1.00	
AC	15.3	15.2	14.2	0.91 (0.27-3.08)	0.885	1.06 (0.28-4.07)	0.932	3.68 (0.80-17.06)	0.095
CC	83.4	83.6	85.1	0.81 (0.23-2.83)	0.742	0.98 (0.25-3.89)	0.974	4.22 (0.85-21.00)	0.078
*P*		0.995	0.581						
*P*_HWE_	0.168	0.174	0.669						
Any C *vs*. AA	98.7	98.8	99.3	0.86 (0.27-2.79)	0.804	1.05 (0.27-4.03)	0.944	3.75 (0.81-17.36)	0.090
Any A *vs*. CC	16.6	16.4	14.9	0.92 (0.65-1.29)	0.615	0.92 (0.65-1.32)	0.659	1.00 (0.59-1.68)	0.991
rs1044250									
TT	0.2	0.3	0.2	1.00		1.00		1.00	
CT	4.2	6.0	6.1	0.87 (0.06-12.52)	0.921	1.34 (0.06-29.65)	0.944	3.16 (0.19-52.71)	0.424
CC	95.6	93.7	93.7	1.19 (0.08-18.09)	0.900	2.21 (0.10-51.65)	0.621	4.00 (0.21-75.39)	0.355
*P*		0.336	0.346						
*P*_HWE_	0.170	0.077	0.556						
Any C *vs*. TT	99.8	99.7	99.8	0.87 (0.06-12.52)	0.921	1.38 (0.06-30.53)	0.840	3.19 (0.19-53.34)	0.419
Any T *vs*. CC	4.4	6.3	6.3	1.35 (0.76-2.42)	0.307	1.61 (0.90-2.90)	0.111	1.14 (0.50-2.62)	0.750
rs2967605									
CC	12.6	17.8	17.9	1.00		1.00		1.00	
CT	48.8	44.2	44.7	0.62 (0.42-0.91)	0.015	0.48 (0.32-0.71)	<0.001	0.72 (0.43-1.27)	0.279
TT	38.6	38.0	37.4	0.78 (0.52-1.16)	0.222	0.62 (0.41-0.93)	0.020	1.05 (0.60-1.86)	0.856
*P*		0.043	0.047						
*P*_HWE_	0.267	0.062	0.102						
Any T *vs*. CC	87.4	82.2	82.1	0.68 (0.47-0.99)	0.043	0.55 (0.38-0.80)	0.020	0.86 (0.52-1.44)	0.570
Any C *vs*. TT	61.4	62.0	62.6	0.88 (0.68-1.16)	0.380	1.05 (0.80-1.37)	0.736	0.76 (0.51-1.14)	0.183

*HWE* Hardy-Weinberg equilibrium, *CAD* coronary artery disease, *IS* ischemic stroke, *AS* angiographic severity of CAD. Adjusted for age, gender, BMI, smoking status, alcohol consumption, hypertension, hyperlipidemia and T2DM

### *ANGPTL4* SNPs and the angiographic severity of CAD

As shown in Table 2, there were no significant associations between the four SNPs and the angiographic severity of CAD in different genetic models (*P >* 0.05).

### Haplotypes and the risk of CAD and IS

A significant LD was noted among the rs4076317, rs7255436 and rs1044250 SNPs (*r*^2^ > 0.80, Table 3). The LD of rs2967605 SNP and other genetic variants was weak in this study. Therefore, the rs2967605 SNP was not included in haplotype analysis. Estimated frequencies of haplotypes derived from three SNPs and their associations with CAD and IS are shown in Table 4. No haplotype of the 3 *ANGPTL4* SNPs was associated with the risk of CAD and IS.

**Table 3:** LD (*r*^2^) between the four *ANGPTL4* SNPs

SNP	rs7255436	rs1044250	rs2967605
rs4076317	0.87 (0.86)	0.90 (0.91)	0.67 (0.63)
rs7255436	-	0.88 (0.85)	0.35 (0.36)
rs1044250	-	-	0.28 (0.32)

LD (*r*^2^) between IS and control was in brackets

**Table 4:** The association between the haplotypes and CAD/IS

Haplotypes	Frequency (%)			CAD		IS	
	Cases (*n* =582)	CAD (*n* =534)	IS (*n* =553)	OR (95% CI)	*P*	OR (95% CI)	*P*
C-A-C	6.5	5.5	4.6	0.83 (0.59-1.18)	0.309	0.69 (0.47-1.00)	0.050
C-C-C	64.1	63.9	64.1	0.99 (0.83-1.17)	0.877	0.99 (0.83-1.18)	0.934
G-C-C	27.0	27.3	28.1	1.01 (0.84-1.22)	0.883	1.06 (0.88-1.28)	0.563

Loci are arranged in the order rs4076317, rs7255436 and rs1044250. Haplotype with frequency less than 3% was pooled and not analyzed

### *ANGPTL4* SNPs and serum lipid levels in the controls

**Fig. 1:**
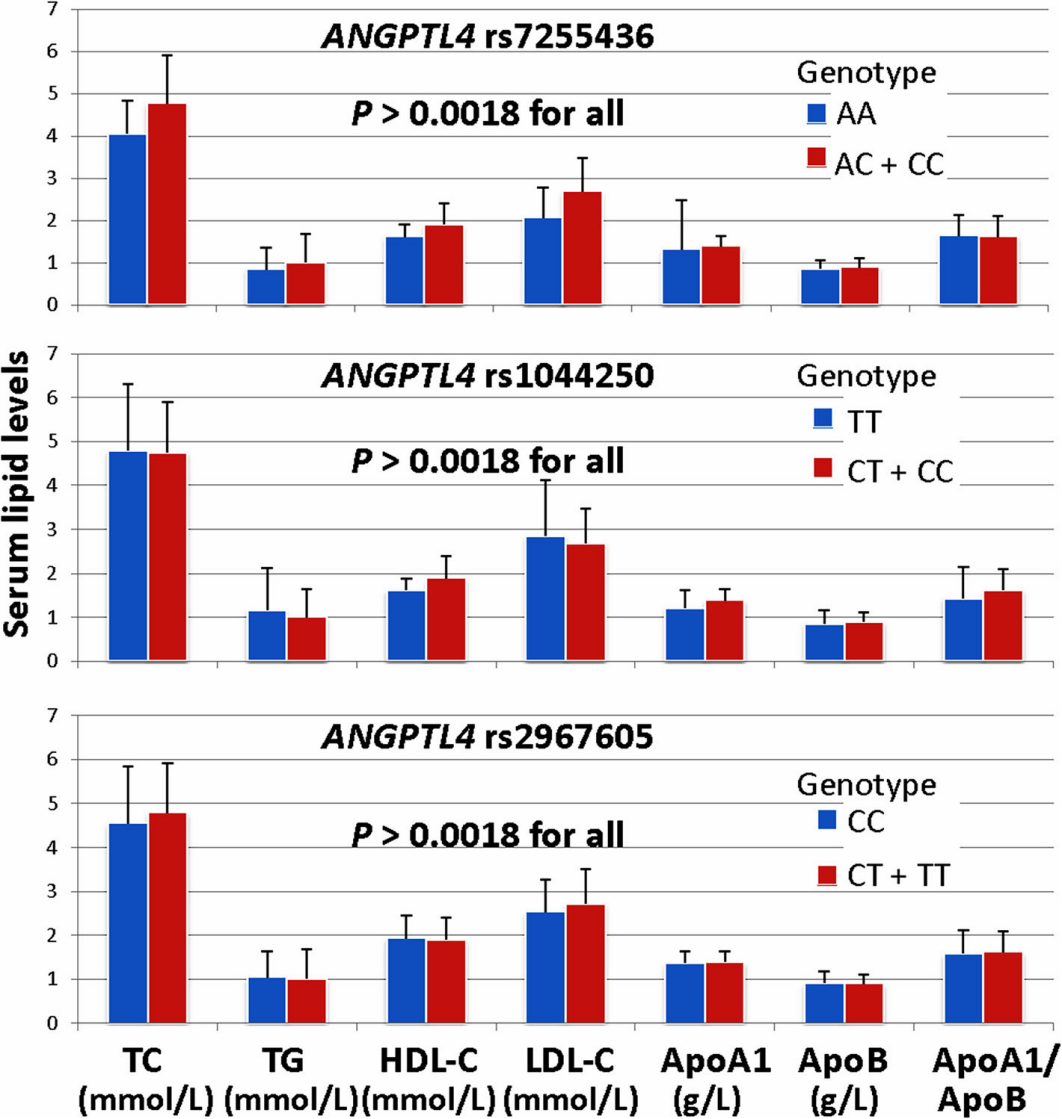
Association of the *ANGPTL4* rs7255436, rs1044250 and rs2967605 SNPs and serum lipid levels in controls. *TC* total cholesterol, *TG* triglyceride, *HDL-C* high-density lipoprotein cholesterol, *LDL-C* low-density lipoprotein cholesterol, *ApoA1* apolipoprotein A1, *ApoB* apolipoprotein B. The value of triglyceride was presented as median (interquartile range), and the difference among or between the genotypes was determined by the Kruskal-Wallis test. *A* value of *P* < 0.0018 was regarded statistically significant after the Bonferroni correction

Significant association was found between the genotypes of the rs4076317 SNP and the levels of TC and LDL-C in the controls (*P* < 0.001; Table 5), the subjects with the CG/CC genotypes in controls had higher TC and LDL-C levels than the subjects with the GG genotype. There were no significant associations between the remaining 3 SNPs and any serum lipid parameters (Fig. 1).

**Table 5:** Association of the *ANGPTL4* rs4076317 SNP and serum lipid levels in the controls

Genotype	n	TC (mmol/L)	TG (mmol/L)	HDL-C (mmol/L)	LDL-C (mmol/L)	ApoA1 (g/L)	ApoB (g/L)	ApoA1/ApoB
GG	40	4.32±1.40	1.21 (0.67)	2.11±0.49	2.34±0.69	1.44±0.24	0.92±0.25	1.68±0.59
CG+CC	509	4.97±1.07	1.00 (0.65)	1.89±0.49	2.77±0.79	1.39±0.24	0.90±0.21	1.62±0.46
*P*		< 0.001	0.024	0.008	< 0.001	0.311	0.998	0.283

*TC* total cholesterol, *TG* triglyceride, *HDL-C* high-density lipoprotein cholesterol, *LDL-C* low-density lipoprotein cholesterol, *ApoA1* apolipoprotein A1, *ApoB* apolipoprotein B. The value of triglyceride was presented as median (interquartile range), and the difference among or between the genotypes was determined by the Kruskal-Wallis test. A value of *P* < 0.0018 was regarded statistically significant after the Bonferroni correction

### *ANGPTL4* rs4076317 SNP and atorvastatin cholesterol-lowering responses

**Fig. 2:**
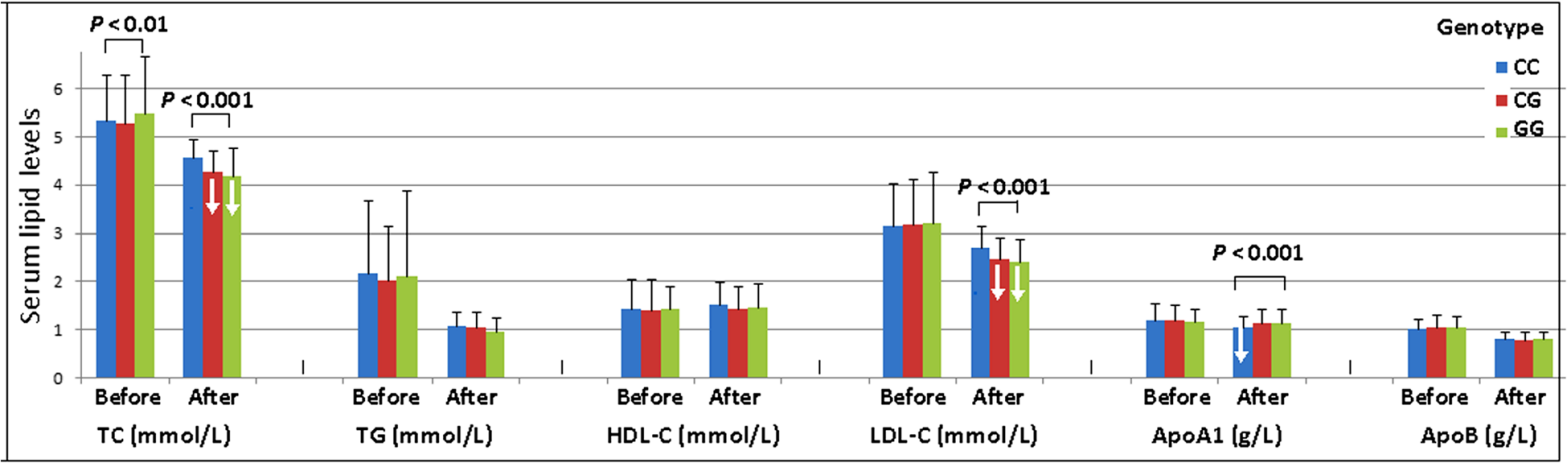
Effects of the ANGPTL4 rs4076317 SNP on serum lipid levels at baseline and response to atorvastatin therapy in hyperlipidemia. TC total cholesterol, TG triglyceride, HDL-C high-density lipoprotein cholesterol, LDL-C low-density lipoprotein cholesterol, ApoA1 apolipoprotein A1, ApoB apolipoprotein B

After 8-week treatment of atorvastatin, the levels of TC, TG, LDL-C, ApoA1 and ApoB were significantly decreased in the total hyperlipidemic patients (*P* < 0.001 for all; Table 6). There was no significant difference in serum HDL-C levels. Subgroup analyses showed that the levels of HDL-C in CAD and IS patients were increased after atorvastatin treatment (*P* < 0.05-0.01). There was no significant difference in serum ApoA1 levels in IS patients and CG/GG genotype individuals after atorvastatin treatment. We also showed that the *ANGPTL4* rs4076317 SNP changed the effects of atorvastatin on serum lipid levels. The subjects with CG/GG genotypes had lower TC and LDL-C levels than the subjects with CC genotype after atorvastatin treatment. However, the subjects with CC genotype had lower ApoA1 levels than the subjects with CG/GG genotypes after atorvastatin treatment (Fig. 2).

**Table 6:** Effects of the *ANGPTL4* rs4076317 SNP on serum lipid levels at baseline and response to atorvastatin therapy in hyperlipidemia

Group	TC (mmol/L)	TG (mmol/L)	HDL-C (mmol/L)	LDL-C (mmol/L)	ApoA1(g/L)	ApoB(g/L)
Total patient (*n* = 724)						
Before	5.33±1.01	2.12±1.39	1.44±0.61	3.17±0.93	1.19±0.35	1.04±0.23
After	4.41±0.46	1.05±0.32	1.48±0.49	2.59±0.46	1.01±0.23	0.81±0.15
*F*	22.305	20.185	1.376	15.042	11.565	22.538
*P*	0.000	0.000	0.169	0.000	0.000	0.000
Control (*n* = 253)						
Before	5.62±0.71	1.74±1.51	1.98±0.54	3.24±0.72	1.47±0.25	1.03±0.19
After	4.67±0.30	0.96±0.31	1.93±0.43	2.61±0.40	1.42±0.21	0.87±0.13
*F*	19.604	8.049	1.152	12.166	2.436	11.055
*P*	0.000	0.000	0.250	0.000	0.015	0.000
CAD (*n* = 248)						
Before	5.14±1.16	2.29±1.18	1.13±0.35	3.08±1.08	1.06±0.35	1.05±0.28
After	4.29±0.48	1.12±0.31	1.20±0.31	2.61±0.52	0.97±0.25	0.78±0.16
*F*	10.663	15.102	2.358	6.175	3.295	13.185
*P*	0.000	0.000	0.019	0.000	0.001	0.000
IS (*n* = 223)						
Before	5.22±1.02	2.36±1.37	1.18±0.49	3.19±0.96	1.03±0.21	1.06±0.22
After	4.26±0.47	1.08±0.32	1.30±0.31	2.53±0.45	1.04±0.21	0.82±0.15
*F*	12.765	13.587	3.091	9.296	0.503	13.460
*P*	0.000	0.000	0.002	0.000	0.615	0.000
CC genotype (*n* = 381)						
Before	5.34±0.95	2.18±1.50	1.45±0.61	3.15±0.89	1.20±0.36	1.03±0.21
After	4.56±0.40	1.07±0.31	1.52±0.49	2.70±0.44	1.04±0.24	0.82±0.15
*F*	14.770	14.145	1.746	8.847	7.218	15.883
*P*	0.000	0.000	0.081	0.000	0.000	0.000
CG genotype (*n* = 284)						
Before	5.29±1.00	2.04±1.11	1.42±0.63	3.18±0.94	1.19±0.34	1.06±0.25
After	4.26±0.45	1.06±0.33	1.43±0.49	2.48±0.44	1.14±0.29	0.80±0.17
*F*	15.829	14.262	0.211	11.366	1.886	14.493
*P*	0.000	0.000	0.833	0.000	0.060	0.000
GG genotype (*n* = 59)						
Before	5.49±1.18	2.12±1.78	1.43±0.48	3.21±1.06	1.16±0.28	1.04±0.26
After	4.18±0.59	0.95±0.31	1.48±0.50	2.40±0.49	1.14±0.29	0.81±0.14
*F*	7.627	4.974	0.554	5.327	0.381	5.983
*P*	0.000	0.000	0.581	0.000	0.704	0.000

*TC* total cholesterol, *TG* triglyceride, *HDL-C* high-density lipoprotein cholesterol, *LDL-C* low-density lipoprotein cholesterol, *ApoA*1 apolipoprotein A1, *ApoB* apolipoprotein B

## Discussion

Several previous GWASes have revealed associations between the *ANGPTL4* SNPs and lipid-related phenotypes or diseases in the European descent. Talmud *et al*. [35] showed significant associations between the rs1044250 (T266M) and lower serum TG and higher HDL-C levels, although this effect was entirely due to the rs116843064 (rare E40K variant). Inconsistently, Staiger *et al*. [10] found no reliable correlations between the rs4076317 and rs1044250 SNPs and fasting TG in Germany White population. Meanwhile, Kathiresan *et al*. [8] revealed that the rs2967605 SNP, which is near *ANGPTL4* and located 30 kbp downstream from *ANGPTL4*, was strongly associated with HDL-C. Dumitrescu *et al*. [40] also reported that the rs2967605 SNP was associated with HDL-C in European Americans, but not in African American, American Indian, and Mexican American/Hispanic. Bryant *et al*. [41] were also unable to replicate this association in non-Hispanic Whites, Hispanics, and African Americans. Parihar *et al*. [42] also could not find the association between the rs2967605 SNP and HDL-C levels in Americans with extreme obesity. In the present study, we detected the association of the four *ANGPTL4* SNPs and serum lipid traits, the risk of CAD and IS in a Southern Chinese Han population. The results showed that the subjects with the rs4076317 CG/CC genotypes had higher TC and LDL-C levels than those with GG genotype. In contrast, no association between the rs4076317 SNP, haplotype carriers and the risk of CAD and IS was observed. In addition, we also found that the rs2967605T allele was associated with a decreased risk of CAD and IS, but no association between the rs2967605 SNP and serum lipid traits was detected.

These findings were different from those of previous studies. The reasons for these different findings remain unclear, one of the important possibilities was different genetic background [8, 41–43]. Somewhat differed with the data from the International HapMap project: the rs4076317G, rs7255436A, rs1044250T and rs2967605C allele frequencies were 83.9%, 61.9%, 31.2% and 78.3% in European descent; respectively. In the current study, the frequencies of rs4076317G, rs7255436A, rs1044250T and rs2967605C allele frequencies in controls were 27.1%, 8.92%, 4.37% and 37.0%, respectively. These results suggest that the *ANGPTL4* variation may have a racial/ethnic-specificity.

Additionally, the underlying molecular mechanisms of the *ANGPTL4* were deserved our concern. Several recent reports have indicated that different ANGPTL4 isoforms might exert diverse physiological functions in different tissues [44]. The N-terminal and full-length ANGPTL4 in the bloodstream inhibited the activity of blood LPL. The C-terminus of ANGPTL4 in endothelial cells, however, had been suggested to regulate vascular permeability and angiogenesis [45]. To our knowledge, angiogenesis is the predominant form of neovascularization in atherosclerosis. Neovascularization in early atherosclerosis is associated with inflammation and lipid deposition, and intraplaque angiogenesis is a risk factor for plaque vulnerability to lead to plaque destabilization and rupture [46, 47]. Consequently, *ANGPTL4* not only regulated TC by inhibiting LPL, but also affected serum lipid levels and arteriosclerosis by other pathways. The impact of *ANGPTL4* on lipids was complex and needed to be further investigated.

Several studies have attempted to address the impact of *ANGPTL4* on atherosclerosis development. Adachi *et al*. [48] showed that fasting and postolive oil-loaded TG levels and atherosclerotic lesion size were largely decreased in *ApoE*(-/-)/*ANGPTL4*(-/-) mice compared with *ApoE*(-/-)/*ANGPTL4*(+/+) mice, and that genetic knockout of *ANGPTL4* protected *ApoE*(-/-) mice against development and progression of atherosclerosis and strongly suppressed the ability of the macrophages to become foam cells *in vitro*. Bouleti *et al*. [49] found that the infarct size was significantly decreased and behavior activity was improved in *ANGPTL4*-treated transient IS model mice, while vascular damage and infarct severity were increased in *ANGPTL4*-deficient mice. In accordance, their results showed that *ANGPTL4* protects not only the global vascular network, but also interendothelial junctions and controls both deleterious inflammatory response and edema, by restricting Src kinase signalling downstream from vascular endothelial growth factor receptor 2 (VEGFR2). Georgiadi *et al*. [16] reported that *ANGPTL4* over-expression reduced lesion area, macrophage content and numbers of monocytes adhering to the endothelium wall. *ANGPTL4* was independently and negatively associated with carotid artery sclerosis measured by 3-T magnetic resonance imaging in subjects with metabolic syndrome and low-grade systemic inflammation. *ANGPTL4* suppresses foam cell formation to reduce atherosclerosis development. In accordance, several reports have also revealed the impact of *ANGPTL4* variations on the development of atherosclerosis [37, 40]. Folsom *et al*. [9] reported that the *ANGPTL4* E40K variant was associated with a decreased risk of CAD. Additionally, He *et al*. [40] found that the carriers of *ANGPTL4* rs4076317GG genotype have lower risk of artery atherosclerotic stroke. Similarly, Muendlein *et al*. [37] also indicated that the rs4076317G allele was a protective effect on future cardiovascular risk, whereas the rs1044250T allele was a risk factor for vascular events. But haplotype defined by the rs1044250T allele provided no additional cardiovascular risk. Inconsistent with previous studies, our research did not discover significant correlations between the rs4076317, rs1044250 SNPs and the risk of CAD and IS. Significant association was found only for the rs2967605 SNP. We just showed that the rs2967605T allele was associated with a decreased risk of CAD and IS. It suggested that the rs2967605 SNP might be functional genetic variant or, alternatively, was in tight linkage with the causative SNP. However, no significant association between the rs2967605 SNP and angiographic severity of CAD was observed, which suggesting its effects are unlikely to be a major pathway for lower CAD and IS risk, although subtle effects cannot be excluded.

Statins are the most commonly used drugs in patients with dyslipidemia and atherosclerotic diseases. They block the conversion of 3-hydroxy-3-methylglutaryl coenzyme A (HMG-CoA) to mevalonate inhibiting cholesterol synthesis in the liver and are effective at reducing atherosclerosis and cardiovascular risks in clinical practice by lowering LDL-C and total TG levels [50]. However, the pharmacodynamic response to statins varies greatly among patients [51]. Statins reduced the risk of complications and death from cardiovascular causes by only approximately one third, leaving the remaining two thirds of patients unprotected [52]. Although the mechanisms have not been fully clarified, genetic polymorphisms may play an important role in individual susceptibility to drug response, including the *ANGPTL4* genetic variants [53–55]. In the present study, we firstly showed that the *ANGPTL4* rs4076317 SNP changed the efficacy of atorvastatin on serum lipid profiles. The subjects with rs4076317CG/GG genotypes had lower TC, LDL-C levels and higher ApoA1 levels than the subjects with CC genotype after atorvastatin treatment. These results suggest that the *ANGPTL4* rs4076317G allele carriers benefited more from atorvastatin therapy than the *ANGPTL4* rs4076317G allele non-carriers in decreasing serum TC and LDL-C levels.

The present study has two potential limitations. First, four SNPs did not cover the whole gene and could not overall elucidate the impact of *ANGPTL4* polymorphisms on serum lipid levels and cardiovascular risk. Second, the variant frequency of the rs7255436 and rs1044250 SNPs was relatively low in the Chinese Han population, which the minor allele frequency was 8.92% and 4.37% respectively. Significant associations between the two SNPs and serum lipid levels and cardiovascular risk could not be realized, probably to the rarity of the variant implicating limited statistical power. Further larger sample studies are needed.

## Conclusions

The *ANGPTL4* rs2967605T allele was associated with a decreased risk of CAD and IS. The subjects with the rs4076317CG/CC genotypes in controls had higher TC and LDL-C levels than the subjects with the GG genotype. The rs4076317G allele carriers benefited more from atorvastatin therapy than the *ANGPTL4* rs4076317G allele non-carriers in decreasing serum TC and LDL-C levels in the Chinese Han population.

## Abbreviations

ADR1: Adiponectin receptor 1
ANCOVA: Analysis of covariance
ANGPTL4: Angiopoietin-like protein 4 gene
Apo: Apolipoprotein
BMI: Body mass index
CAD: coronary artery disease
CI: Confidence interval
DNA: Deoxyribonucleic acid
GWAS: genome-wide association study
HDL-C: High-density lipoprotein cholesterol
HMG-CoA: 3-hydroxy-3-methylglutaryl coenzyme A
HWE: Hardy-Weinberg equilibrium
IS: ischemic stroke
LD: Linkage disequiblirium
LDL-C: Low-density lipoprotein cholesterol
LPL: Lipoprotein lipase
MAF: Minor allele frequency
OR: Odds ratio
PPAR: Peroxisome proliferator-activated receptor
RCT: Reverse cholesterol transport
SNPs: Single nucleotide polymorphisms
SR-BI I: Scavenger receptor class B type I
T2DM: Type 2 diabetes mellitus
TC: Total cholesterol
TG: Triglyceride
TOAST: Trial of Org 10712 in Acute Stroke Treatment
VEGFR2: Vascular endothelial growth factor receptor 2
VLDLs: Very low-density lipoproteins
